# Computer game-based and traditional learning method: a comparison regarding students’ knowledge retention

**DOI:** 10.1186/1472-6920-13-30

**Published:** 2013-02-25

**Authors:** Silmara Rondon, Fernanda Chiarion Sassi, Claudia Regina Furquim de Andrade

**Affiliations:** 1 Department of Physiotherapy, Speech-Language and Hearing Sciences and Occupational Therapy, School of Medicine, University of Sao Paulo, Sao Paulo, Brazil; 2 Rua Cipotânea, 51 – Cidade Universitária, CEP: 05360-160, São Paulo/S.P, Brazil

**Keywords:** Speech, Language and hearing sciences, Anatomy, Physiology, Stomatognathic system, Learning, Computer-assisted instruction

## Abstract

**Background:**

Educational computer games are examples of computer-assisted learning objects, representing an educational strategy of growing interest. Given the changes in the digital world over the last decades, students of the current generation expect technology to be used in advancing their learning requiring a need to change traditional passive learning methodologies to an active multisensory experimental learning methodology. The objective of this study was to compare a computer game-based learning method with a traditional learning method, regarding learning gains and knowledge retention, as means of teaching head and neck Anatomy and Physiology to Speech-Language and Hearing pathology undergraduate students.

**Methods:**

Students were randomized to participate to one of the learning methods and the data analyst was blinded to which method of learning the students had received. Students’ prior knowledge (i.e. before undergoing the learning method), short-term knowledge retention and long-term knowledge retention (i.e. six months after undergoing the learning method) were assessed with a multiple choice questionnaire. Students’ performance was compared considering the three moments of assessment for both for the mean total score and for separated mean scores for Anatomy questions and for Physiology questions.

**Results:**

Students that received the game-based method performed better in the pos-test assessment only when considering the Anatomy questions section. Students that received the traditional lecture performed better in both post-test and long-term post-test when considering the Anatomy and Physiology questions.

**Conclusions:**

The game-based learning method is comparable to the traditional learning method in general and in short-term gains, while the traditional lecture still seems to be more effective to improve students’ short and long-term knowledge retention.

## Background

Computer-Assisted Instruction (CAI) is an additional modality of teaching methods that incorporate multimedia to present knowledge [1]. Features such as the functionality to incorporate multimedia, to present knowledge in a setting similar to that in which it will be used [2], to provide access to learning materials in a time and place convenient for the learner [3], and to provide interactive feedback critical for self-assessment [4] should be considered when using CAI, especially in medical/health education. Given the changes in the digital world over the last decades, students of the current generation expect technology to be used in advancing their learning requiring a need to change traditional passive learning methodologies to an active multisensory experimental learning methodology [5,6]. However, one should always have in mind that it is important to consider that the quality of the teaching provided by the CAI remains more important to both student satisfaction and learning than technology [7].

Knowledge related to head and neck Anatomy and Physiology is complex and extremely important for guidance in the processes of assessment, diagnosis and intervention in the field of the Speech-Language and Hearing Science [8-10]. When these concepts are well assimilated in the early stages of formation it is possible to avoid situations where the student only realizes the importance of these concepts when he is put ahead of his first patient [11]. Well-designed CAI has been shown to be effective in producing lasting clinical skills in Health Sciences education, although literature provides little guidance on the relative advantages of specific instructional and technical design features to maximize learning [12-15].

With the advance in information technology, computer-assisted learning environments and objects have been incorporated to Anatomy and Physiology laboratories and classes to enhance learning [16,17]. Ohrn, van Oostrom and van Meurs [18] performed a comparison of traditional textbook and interactive computer learning of neuromuscular blockade among first-year anesthesia residents. The results presented by the authors indicated that the improvement in test scores was significantly greater for the computer group than for the textbook group. Also, differences in the enjoyment and quantity learned rankings of the two groups were found to be significant in favor of the computer program. In a different study, Goldberg and McKhann [17] investigated the performance of students in a virtual learning environment to learning topics in Neuroscience and compared with that of students in a conventional lecture. The results consistently demonstrated higher test scores in the virtual learning environment as opposed to the conventional lecture, regardless of the time in the semester at which the knowledge tests were given.

Studies have shown that learning objects that provide educational alternatives for reasoning involving a problem solving situation (i.e. considering the student’s prior knowledge and cognitive architecture) are more appropriate for learning [19-23]. According to these studies, this occurs because learning methods that use these types of objects may reduce the working memory cognitive load and therefore facilitate the learning process. Educational computer games are examples of this type of learning object [24,25] which represents an educational strategy of growing interest [26]. These games have characteristics related to problem solving, providing the student with different possibilities to elaborate strategies and to achieve their predetermined goals [27].

Prensky [28] stressed that the key to learn about the effectiveness of digital/computer games lay in their design. The design of computer game-based learning methods should include clear rules and goals, and most importantly, the game must be fun to play and gain values [29]. Computer games should contain questions with complexities that favors students’ performances and their learning process [30,31]. Additionally, good quality games should give the player feedback about his/her actions and new problems to be solved [32].

Students can develop cognitive skills such as memory, attention and critical thinking through the use of computer games, besides being able to elaborate and confirm their hypothesis [32-35]. In addition, students can construct their knowledge in a more integrative way (i.e. integrating their knowledge with their actions), with higher motivation to learn [27,29,36-38].

In response to the lack of empirical studies examining the differential effects of computer games on the academic performance of diverse learners, and the lack of consensus that had not been reached on the effects of computer games on student achievement, Kim and Chang [38] empirically examined the effects of math computer games on the math performance of 4^th^ grades with focused attention to differential effects for gender and linguistic groups. The results showed that English-speaking students who played computer math games in school everyday displayed significant lower math achievement than those who never played. Contrastingly, positive effects of daily computer use were noted among male students whose first language was other than English. Male language minority students who daily played computer games in math demonstrated higher math performance scores compared with their male English-speaking counterparts who never played.

Positive results have been found and different types of games have been incorporated in Higher Education, including Health Sciences courses [39]. A pioneering study indicated that medical students who used a computer game about the administration of a particular drug, presented higher percentages of correct decision making actions related to the covered topic [40]. Another study, developed in Civil Engineering area, identified that playing an educational computer game leads to equivalent learning results as participating of a traditional method and the game environment leads to increasing motivation – the learner plays the game again [27]. The study of Kanthan and Senger [41] provided insight that specially designed content-relevant digital games can be used as an additional, e-teaching/learning resource for the teaching of pathology in undergraduate medical education; improve academic performance on examination test scores; increase student engagement, promote student satisfaction and reduce student stress; and foster an improved, facilitated, fun, nonthreatening, extended study learner environment.

Games may have potential in improving learners’ knowledge, skills, attitudes and behaviours [42]. Therefore, computer simulation games, for instance, may have multiple effects on problem solving with computer programs. Kiili [43] has argued that games can be a vehicle for engaging students in a “flow”. Flow is a consciousness state during which an individual is in control of his actions, and in which there is little distinction of self and environment, between stimulus and response [44,45]. Liu, Cheng, Tsai and Huang [37] considered flow as an useful construct for improving problem solving. These authors analyzed the feedback and problem solving of undergraduate students in a simulation game, designed to assist them to learn computational problem solving. It was found that students when learning computational problem solving with the game were more likely to perceive a flow learning experience than in traditional lectures. In particular, the results of the study indicated a close association between the students’ learning experience and their problem solving strategies.

Although evidence exists on the benefits of using games (including computer games) in the education of Health Sciences students, more studies are necessary about how to conduct pre and post intervention assessments (i.e. conducting a baseline assessment in addition to the post-test assessment), considering educational and clinical aspects [39]. The main objective of the assessments should be to measure the results of using computer games in terms of learning performance and knowledge retention [39]. Until this date, there are no related studies in the field of the Speech-Language and Hearing Science about the use of computer game-based learning methods.

The purpose of this study was to compare a computer game-based learning method with a traditional learning method, regarding learning gains and knowledge retention, as means of teaching head and neck Anatomy and Physiology to Speech-Language and Hearing pathology undergraduate students. We set the following hypothesis for our present study:

1) The game will be as effective as traditional learning method concerning the gain of knowledge that the game is supposed to reinforce and integrate, if measured immediately after the conclusion of the exposure.

2) The long-term knowledge retention will be higher in the game group.

## Methods

This study was conducted with second-year Speech-Language and Hearing Science students of the School of Medicine of University of São Paulo, who were undertaking a head and neck Anatomy and Physiology class. This included weekly teaching sessions and a study schedule developed in the classroom environment.

To be included in the study, students should have successfully completed the introductory classes of Anatomy and Physiology and had to be proficient in English reading comprehension.

Each student was randomly allocated in one of two groups: Group I (GI)– 15 students who were submitted to the computer game-based learning method (CGBLM); Group II (GII) – 14 students who were submitted to the traditional learning method (TLM). Both methods had the same duration (one-hour, once a week), and were delivered by the same tutor. The tutor was blinded to the random allocation process.

The study design was approved by the Ethics Committee for the Analysis of Research Projects (CEP FMUSP no. 080/10). Prior to their enrolment, all participants were informed of the purpose and procedures, after which all gave informed consent.

The application of the learning methods was developed over nine weeks. The content of the learning methods was the same for both groups. For GI, the quiz section of the software *Anatesse 2.0*[45] was used. For GII, short scientific texts related to the topics discussed in class were used. The topics selected for both learning methods were the same.

In the CGBLM, a notebook integrated with a multimedia projector was used to play the quiz in *Anatesse 2.0. Anatesse 2.0* is interactive student learning software containing animations, chapter support, and self-study quizzes to aid learning and augment understanding of anatomy and physiology of the speech, language, hearing, and swallowing mechanisms which integrate a section of *The Electronic Classroom Manager to accompany the book Anatomy & Physiology for Speech, Language and Hearing, Third Edition.* This material is presented in a CD-ROM format and it is divided into these major sections: the *ExamView® Computerized Test Bank* contains over 1000 questions. These questions can be used for the teacher to create their own review materials or tests; the *Instructor’s Manual* includes a wide variety of valuable resources to help with planning the course and implementing activities by chapter for classroom use – the availability of this manual in an electronic format increases its ease of use and value as a teaching resource; an *Image Library* containing electronic versions of some images from the book that can be used to develop handouts; and *Anatesse 2.0*.

For our study, only the quiz section was used. The quiz section (i.e. computer game) contains multiple-choice questions, involving text and figures, and is divided by topic (e.g. bones of the head, muscles of the face, muscles of the tongue etc.). Feedback was given by the software immediately after each answer. If the answer was correct, a picture representing a happy face appeared on screen; if the answer was incorrect, a picture representing a sad face was shown. Students were given a total score at the end of each quiz topic. Each week one quiz topic was selected, according to what was covered in class. During each week, one student was chosen by his/her classmates to operate the software system (i.e. register answers after group discussion). There was only one computer for the whole group. Each quiz topic, containing several questions, was played twice. The first time students answered the questions, only a happy or sad face feedback was given. During the second round, the correct answer was given automatically, in order to enhance the feedback of performance.

In the TLM students were given short scientific texts, one per week, containing relevant information and pictures. Students were instructed to perform their study in the same way they were used to when studying at home. This could be done individually or in groups.

To assess students’ prior knowledge, short-term knowledge retention and long-term knowledge retention, a fifty multiple choice questionnaire (containing four alternatives each) was developed and applied in three different points: before the application of the learning methods (pre-test), immediately after the class conclusion(post-test) and six months after class conclusion (long-term post-test). The questionnaire was specifically designed for this research, since there are no standardized materials for testing knowledge about head and neck Anatomy and Physiology in the field of the Speech-Language and Hearing Science. Questions were classified as being related to Anatomy or to Physiology. To ensure the quality and relevance of the questions, the questionnaire was submitted to two independent judges, speech-language pathologists, with a Ph.D in the covered topics. Interjudge reliability was of .96, representing an excellent level of agreement.

The performance between the groups was compared, considering the three points of assessment. Comparisons were made using both the total number of correct answers, as well as the number of correct answers regarding Anatomy and Physiology.

### Data analysis

One-way ANOVA with two factors was used to perform between group comparisons (i.e. for the mean total score and for the mean scores obtained in each section of the questionnaire, in the three moments of assessment) [46]. Bonferroni correction for multiple comparisons was used to ensure .05 level of significance and to verify where the significant differences occurred. One-way ANOVA with one factor was used to perform within group comparisons [46].

## Results

Twenty nine students were randomized to an intervention - one student from GI was excluded for not having completed the multiple choice questionnaire for the long-term knowledge retention assessment; three students from GII were excluded for not having completed the multiple choice questionnaire for the prior knowledge assessment. Thirteen students played the computer game and twelve students were given thetraditional lecture.

Most students were female (92.0%), with mean age of 23.0 years (SD = 6.2). There was no statistically significant difference for age when comparing both groups (Mann–Whitney test, *p* = .062). Our study did not consider gender aspect as a variable of investigation. It is important to highlight at this moment that in Brazil, approximately 95% of the Speech-Language and Hearing Science undergraduate students are female. For this reason, it would not to be possible to have a balanced sample containing an equivalent number of males and females.

Descriptive analyses of the students’ scores obtained in the knowledge assessment according to the group, the category of questions and the point of assessment are presented in Table 1.

**Table 1:** Descriptive statistics of knowledge assessment

**Area of knowledge**	**Moment**	**GI (n = 13)**		**GII (n = 12)**	
		**Mean (SD)**	**Median (min-max)**	**Mean (SD)**	**Median (min-max)**
**ANATOMY**	Pre test	16.6(2.5)	18.0(12 – 19)	17.6(2.1)	17.5(14 – 20)
	Post test	19.1(2.4)	19.0(14 – 22)	21.3(1.7)	22.0(18 – 24)
	Lt post test	19.5(3.9)	21.0(14 – 26)	19.3(3.5)	19.5(13 – 25)
**PHYSIOLOGY**	Pre test	11.2(2.6)	11.0(7 – 16)	12.3(3.3)	12.5(7 – 18)
	Post test	14.1(1.8)	14.0(11 – 17)	14.8(2.2)	15.0(11 – 18)
	Lt post test	13.5(2.9)	14.0(7 – 18)	13.4(1.6)	13.5(10 – 16)

Legend: *n* – number of students; *SD* – standard deviation; *min* – minimum; *max* – maximum; *Lt post test* – long-term post test.

**Figure 1:**
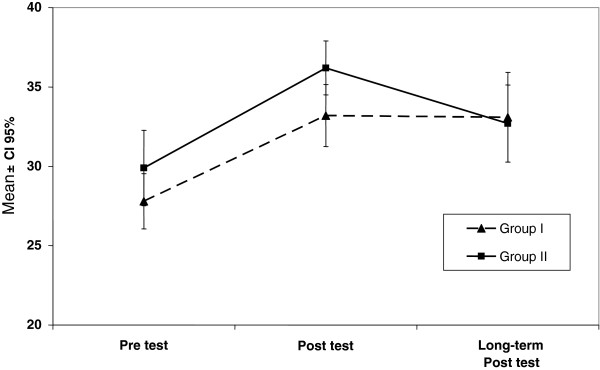
Total results obtained (mean ± CI 95%) according to learning method and moment of assessment.

Considering the between group analysis, a statistically significant difference was observed for both groups when comparing results obtained in the three points of assessment (*p* < .001). However, there were no significant differences between the groups related to the learning methods (*p* = .176), neither in terms of the mean scores obtained in the knowledge questionnaire, nor when considering the three points of assessment (*p* = .699) (Figure 1).

The Tukey test indicated that, for both groups, pre-test scores were significantly lower when compared to the post-test and the long-term post-test. Although it is possible to observe a trend towards a better long-term retention for GII (Figure 1), no significant difference was observed when comparing the performance in the post-test and long-term post-test (Table 2).

**Table 2:** Between group comparisons regarding total scores in the three points of assessment

**Point of assessment**	***p *value**
Pre-test × Post-test	**<0.001**
Pre-test × Long-term post-test	**0.002**
Post test × Long-term post test	0.239

There was a statistically significant difference in the results obtained along the research period for the Anatomy (*p* < 0.001) and Physiology (*p* = .001) questions. However no significant difference between learning methods was observed, even when considering the comparison group x point of assessment along the complete period of the research (Table 3).

**Table 3:** Between group comparisons regarding the total score in anatomy and physiology questions and the point of assessment

**Comparisons**	**Anatomy questions (*p *value)**	**Physiology questions (*p *value)**
GI × GII	0.247	0.354
Group × Point of assessment	0.161	0.601

Within group comparisons indicated overall significant differences for both groups when comparing the scores obtained in the pre and post-test. Comparison between pre and long-term post-test scores indicated significant differences only for GII. No significant difference was observed between the students’ total scores obtained in the post-test and in the long-term post-test (Table 4).

**Table 4:** Within group comparisons regarding the total scores according to the point of assessment

**Point of assessment**	**GI (*p *value)**	**GII (*p *value)**
Pre-test × Post-test	**0.002**	**0.004**
Pre-test × Long-term post-test	0.092	**0.021**
Post test × Long-term post-test	0.111	>0.999

Results obtained from Anatomy section of questionnaire indicated that GI obtained better scores in the comparison between pre and post-test (*p* < .001) and GII obtained better scores in the comparison between pre and long-term post-test (*p* = .042). Results obtained from the scores related to the Physiology section of the questionnaire indicated that only GII presented significant difference when comparing pre and post-test scores (GI – *p* = .064; GII – *p* = .019).

## Discussion

In the present study we compared a computer game-based learning method with a traditional lecture as means of teaching head and neck Anatomy and Physiology to second-year Speech-Language and Hearing Pathology undergraduate students. Both methods were compared considering students’ learning gains and knowledge retention. The results showed that the CGBLM is comparable to the TLM concerning knowledge gains when measured immediately after the learning method application. This result agrees with the results of previous studies that investigated the effects of educational computer games in students’ knowledge reinforced and integrated by a computer game, as well as knowledge learnt during expository lectures but not strengthened by a computer game [27,47].

There are many studies regarding the impact of using different computer games (i.e. in terms of the complexity and the type of games) in students’ motivation and engagement to learn [27,37,39,41]. However, the data about knowledge retention are thin [36]. Studies have shown that students demonstrate a positive effect size regarding knowledge retention from computer game-playing, when assessed immediately after or up to one month after game exposure [41,48]. In the present study, the same result was found in terms of students’ short-term knowledge retention, confirming the first hypothesis of the study.

Egenfeldt-Nielsen [49] found a small gain in terms of long-term knowledge retention when students were assessed five months after game exposure. In our study, only students in the TLM group presented gains in terms of long-term knowledge retention (i.e. within group analysis), assessed six months after the application of the learning method, when comparing pre and long term post-test performances. Although the literature reports that the use of computer games increases the engagement and the motivation to learn [27], in some situations people still seem to be more comfortable with printed out texts. The reading activity gives students the possibility to pause, resume and cover the ideas presented [50]; during the computer game-playing the students may have other distractions [51]. In our study, during the computer game-playing, the feedback was given immediately after each question (i.e. if answer was correct or not), in order to reinforce the content presented. However, the order in which the questions were presented in the screen was determined by the software system, and it was not possible for students to go back and review their answers and contents of interest. In our study, the possibility to review information could only be done by the students in the TLM group.

Our study had a few limitations. We have to consider that the group of students who underwent the CGBLM faced as a limitation the existence of only one computer for the whole group. This may have interfered in the students’ interest in the computer game or even in their attention span.In general, studies that have presented good results for computer game-based learning methods refer to the use of one computer per student [27,37,38,41,48]. Also our study did not investigate differences in performance related to gender. Previous researches suggest that is almost impossible to separate students’ experience with video games from gender issues as male not tend only tend to play games more often, but they also play different types of games [52,53] and they hold significant attitudes toward the use of video games [54]. However, some studies indicated that games can be equally effective and motivating for both male and female. These studies suggest that the impact of gender on acceptance tends to disappear during the implementation phase [55,56]. In a recent study regarding medical student attitudes toward video games and related new media technologies in medical education [57] the results indicated that men and women agreed that they were most inclined to use multiplayer simulations if they were fun, and if they helped to develop skill in patient interactions. Significant gender dissonance was observed over types of favorite games, the educational value of video games, and the desire to participate in games that realistically replicated the experience of clinical practice. This point should be considered in future investigations.

Further studies need to be carried out considering other factors that interfere with learning through computer games, as the motivation for learning and the type of computer game used, according to their educational objectives. As indicated by the literature, these variables can have an influence in the performance of students [27,41,47], not only in terms of knowledge gains but also when considering clinical practice (i.e. in the case of the Health Sciences) [39].

The use of computer games in the classroom environment is a novel proposal in in the field of the Speech-Language and Hearing Sciences. Using computer games as a complementary educational resource in Higher Education is an innovative proposal, and many challenges must be addressed, particularly regarding the development and the application of a type of learning object whose use is still far from the educator reality [58], especially in Higher Education [59].

With the increasing development of educational computer games and their use in Higher Education, further studies are necessary for rigorous evaluation of the computer games effectiveness in improving educational and clinical outcomes [26]. This should be performed by developing methods for the assessment of students' knowledge retention [37,41].

The present study represents a first initiative to investigate the use of computer games in the field of Speech-Language and Hearing. The follow up proposal for this study is to increase sample size, to assess learning motivation when using computer gamesand to investigate the effects of computer games on clinical reasoning and decision making in the field of the Speech-Language and Hearing Sciences.

## Conclusion

The results of the present study showed that the computer game-based learning method is comparable to the traditional learning method concerning knowledge gains when measured immediately after the learning method application (short-term knowledge retention). Moreover, the traditional lecture seems to be more effective to improve students’ long-term knowledge retention. In general, this finding is more important than the first one because long-term effects of curricular education are more crucial than short-term effects.

It is important to point that the results presented in this study should not undermine the use of computer games in classroom environment even in Higher Education. Rather, it helps reinforce the critical need for further research aimed at assessing the educational value of computer games in students’ learning and knowledge retention.

## Competing interest

The authors declare that they have no competing interest.

## Authors’ contribution

SR contributed to data collection and analysis, to the interpretation of the results, to the manuscript writing and provided substantial scientific contribution. FCS contributed to the interpretation of the results, to the manuscript writing and provided scientific contribution. CRFDA contributed to the research and experimental design. All authors read and approved the final manuscript.

## Authors’ information

Silmara Rondon is a speech pathologist at the Department of Physiotherapy, Speech-language and Hearing Sciences and Occupational Therapy. School of Medicine, University of São Paulo, Brazil.

Fernanda Chiarion Sassi has a Ph.D. in Science and is a speech pathologist at the Department of Physiotherapy, Speech-language and Hearing Sciences and Occupational Therapy. School of Medicine, University of São Paulo, Brazil.

Claudia Regina Furquim de Andrade is a Full Professor at the Department of Physiotherapy, Speech-language and Hearing Sciences and Occupational Therapy. School of Medicine, University of São Paulo, Brazil.

## Pre-publication history

The pre-publication history for this paper can be accessed here:

http://www.biomedcentral.com/1472-6920/13/30/prepub
